# Combined Radiomic and Visual Assessment for Improved Detection of Lung Adenocarcinoma Invasiveness on Computed Tomography Scans: A Multi-Institutional Study

**DOI:** 10.3389/fonc.2022.902056

**Published:** 2022-05-30

**Authors:** Pranjal Vaidya, Kaustav Bera, Philip A. Linden, Amit Gupta, Prabhakar Shantha Rajiah, David R. Jones, Matthew Bott, Harvey Pass, Robert Gilkeson, Frank Jacono, Kevin Li-Chun Hsieh, Gong-Yau Lan, Vamsidhar Velcheti, Anant Madabhushi

**Affiliations:** ^1^ Department of Biomedical Engineering, Case Western Reserve University, Cleveland, OH, United States; ^2^ Department of Radiology, University Hospitals Cleveland Medical Center, Cleveland, OH, United States; ^3^ Department of Surgery, Division of Thoracic and Esophageal Surgery, University Hospitals Cleveland Medical Center, Cleveland, OH, United States; ^4^ Department of Radiology, Mayo Clinic, Rochester, MN, United States; ^5^ Department of Surgery, Memorial Sloan Kettering Cancer Center, New York, NY, United States; ^6^ Department of Cardiothoracic Surgery, New York University (NYU) Langone Health, New York, NY, United States; ^7^ Division of Pulmonary Medicine, Louis Stokes VA Medical Center, Cleveland, OH, United States; ^8^ Department of Radiology, School of Medicine, College of Medicine, Taipei Medical University and Taipei Medical University Hospital, Taipei, Taiwan; ^9^ New York University (NYU) Langone Perlmutter Cancer Center, New York, NY, United States; ^10^ Louis Stokes Cleveland Veterans Administration Medical Center, Cleveland, OH, United States

**Keywords:** radiomics, minimally invasive adenocarcinoma (MIA), ct scan (CT), integrated model analysis, invasive adenocarcinoma (IA), radiologists interpretation

## Abstract

**Objective:**

The timing and nature of surgical intervention for semisolid abnormalities are dependent upon distinguishing between adenocarcinoma-*in-situ* (AIS), minimally invasive adenocarcinoma (MIA), and invasive adenocarcinoma (INV). We sought to develop and evaluate a quantitative imaging method to determine invasiveness of small, ground-glass lesions on computed tomography (CT) chest scans.

**Methods:**

The study comprised 268 patients from 4 institutions with resected (<=3 cm) semisolid lesions with confirmed histopathological diagnosis of MIA/AIS or INV. A total of 248 radiomic texture features from within the tumor nodule (intratumoral) and adjacent to the nodule (peritumoral) were extracted from manually annotated lung nodules of chest CT scans. The datasets were randomly divided, with 40% of patients used for training and 60% used for testing the machine classifier (Training D_Train_, N=106; Testing, D_Test,_ N=162).

**Results:**

The top five radiomic stable features included four intratumoral (Laws and Haralick feature families) and one peritumoral feature within 3 to 6 mm of the nodule (CoLlAGe feature family), which successfully differentiated INV from MIA/AIS nodules with an AUC of 0.917 [0.867-0.967] on D_Train_ and 0.863 [0.79-0.931] on D_Test_. The radiomics model successfully differentiated INV from MIA cases (<1 cm AUC: 0.76 [0.53-0.98], 1-2 cm AUC: 0.92 [0.85-0.98], 2-3 cm AUC: 0.95 [0.88-1]). The final integrated model combining the classifier with the radiologists’ score gave the best AUC on D_Test_ (AUC=0.909, p<0.001).

**Conclusions:**

Addition of advanced image analysis *via* radiomics to the routine visual assessment of CT scans help better differentiate adenocarcinoma subtypes and can aid in clinical decision making. Further prospective validation in this direction is warranted.

## Introduction

Lung cancer is the leading cause of cancer related deaths in the world. Adenocarcinoma is the most common lung cancer histologic type (1). With the increase in diagnostic imaging methods such as low-dose chest CT screening, there has been an increase in the detection of lung cancers at earlier stages often presenting as small solid/semisolid nodules or ground-glass opacities (GGOs) (2–4). The new IASLC guidelines (5) and the AJCC-defined 8th edition staging guidelines (6), along with the WHO classification of adenocarcinomas (7), have divided the adenocarcinoma into three broad categories: preinvasive adenocarcinoma [including adenocarcinoma *in situ* (AIS)], minimally invasive adenocarcinomas (MIA) and invasive adenocarcinoma (INV) (8). Histopathologically, lepidic growth (defined as growth along the alveolar walls) is a hallmark of non-invasive lesions (8). An invasive component in the new classification system is defined as either any cellular histologic subtype other than lepidic or invasion of malignant cells into myofibroblastic stroma (9). Lepidic cancers are observed to follow an orderly progression from the AIS to MIA before becoming INV (10).

Outcomes of adenocarcinomas following surgical resection are dependent on the initial stage. Resected stage IA non-small cell lung cancer (NSCLC) has a five-year overall survival rate of about 75% (11). In comparison, the five-year disease-specific survival rate for resected MIA is nearly 100% (12). The surgical approach and extent of lung resection for these lung nodules can be dictated by the adenocarcinoma histologic subtype (13). Sublobar resection can produce equivalent results to lobectomy in patients with non- or minimally invasive adenocarcinomas, with the benefit of preservation of lung parenchyma and potential eligibility for repeat resection in the case of subsequent primary tumor.

At present, there are no definite radiographic biomarkers to identify the extent of invasion prior to surgical resection. Although the invasive portion of the cancer is typically solid and non-invasive (lepidic portion) is ground glass in appearance on the CT scan, there is substantial overlap in the imaging findings between different subcategories. Furthermore, traditional CT scan evaluation can be subjective, and interpretations tend to vary widely depending on the experience of the reading radiologist (14). This coupled with other variables such as scan parameters, slice thickness, etc. limits reliable differentiation on routine radiologic assessment. Fine needle aspiration and imaging is inaccurate in determining the degree of invasion (15). Hence, there is a critical need to create an accurate model to non-invasively assess the level of invasion on imaging in these early-stage adenocarcinomas prior to surgical resection.

Radiomic textural features represent high-throughput quantitative imaging data extracted from radiographic scans to investigate subtle patterns within a region of interest (ROI) (16). These textural patterns extracted from inside and outside the nodule have been shown to have diagnostic, prognostic, and predictive utility in the lung cancer domain (17). These features are known to capture the underlying tumor biology and morphology of the tissue (18, 19). There have been previous attempts at identifying the level of invasion using radiomic features, but most of them focus on radiomic textural analysis solely within the tumor (20, 21). The peritumoral microenvironment has emerged as a promising candidate location for identifying the level of invasion, although it has been relatively unexplored (22).

In this study, we constructed a non-invasive radiographic biomarker based on baseline chest CT scan-guided radiomics to distinguish MIA from INV for stage I NSCLC patients with tumor diameter less than 3 cm. We evaluated these radiomics features *via* supervised and unsupervised approaches to identify specific patterns associated with INV and MIA nodules. We also divided patients into different subgroups based on the diameter of the nodule and evaluated classifier performance within nodules with different sizes. Finally, we compared our model with the performance of two radiologists and integrated the radiologists’ score with the corresponding machine classifier performance to assess combined human and machine classification performance.

## Materials and Methods

### Study Population

We performed a retrospective, multi-cohort study of patients with resected MIA and stage 1A INV cases. A total of 268 patients from four different institutions were included in the study, all of whom had baseline (pre-treatment) CT scans. Based on our inclusion criteria, we selected a cohort of patients who had tumor size less than or equal to 3 cm with a special focus on a subset of 1 to 2 cm nodules.

The patients were randomly divided into training (D_Train_=40%) and validation (D_Test_=60%) cohorts. The D_Train_ was selected to keep the same number of invasive and non-invasive lesions for training the model.

### Procedure

#### CT Segmentation and Radiomic Textural Feature Extraction

The index pulmonary lesions on these baseline CT scans were annotated using a freehand tool on 3D slicer software by an expert radiologist. The details regarding the CT scan parameters are listed in **Appendix 1**.

After the tumor was annotated, the area of the nodule was calculated using MATLAB 2015. The tumor area was calculated upon identification of the CT slice with the largest tumor region and was used for subgroup analysis and for creating a combined radiomics area-based model.

These annotated nodules were used to extract the intra- and peri-tumoral texture features. The peri-tumoral compartment around the nodule was defined *via* quantitative morphological operations (dilation) as a region extending radially from the nodule boundary up to roughly 15 mm, since a resection margin larger than 15 mm for lung nodules is considered not to confer additional benefit in terms of invasive lesions. The program was modified to eliminate skin, air, or fat when the mask was extended. Radiomic peritumoral features were extracted in an annular ring-shaped fashion. Five annular rings peritumorally were analyzed, each with 3-mm increments leading up to a maximum radius of 15 mm from the nodule periphery.

The details regarding extracted radiomic features are provided in **Appendix 1**. Haralick and Collage features are based on constructing a gray-level co-occurrence matrix and are known to capture the general disorganized and chaotic microarchitecture of the annotated region of interest (23, 24). The Laws and Laplace features focus on the high-frequency content of the image, focusing on the boundary of the ROI (25). Gabor features are wavelet-based features (26).

#### Classifier Construction

All patients included in the study were divided into two groups: pre-invasive/minimally invasive lesion group (AIS, MIA) and frank invasive group (invasive pulmonary adenocarcinoma [IPA]). These two groups were used as a clinical endpoint for the classification problem.

First, all the radiomic features were analyzed using an unsupervised clustering approach to evaluate the ability of the radiomic features to differentiate the two different diagnostic categories blinded to prior pathology results or clinical outcome. First, the PCA was used on an entire feature pool and the top three principal components were used within K-Means clustering analysis. In addition, the hierarchical clustering was performed on an entire cohort.

Next, a supervised machine learning based logistic regression classifier, M_R_, was constructed using the top selected features from the training cohort, D_Train,_ and then was validated on an independent and blinded validation set D_Test_. Further, D_Test_ was divided into 3 different subsets based on the nodule size (less than 1 mm, 1 mm-2 mm, 2 mm-3 mm) and the performance of the model was observed on these various subgroups defined using nodule sizes.

Next, another supervised machine classifier model was constructed using the tumor areas, M_A_, and further integrated with radiomic features to construct the combined tumor area-radiomics based model (M_R+A_).

#### Human Reader Experiment

The patients from D_Test_ were individually assessed by two radiologists with 12 and 21 years of experience, respectively, being blinded to the ground truth pathologic diagnosis of the nodules. The two readers scored each tumor from 1 to 3; 1 suggesting the nodule was MIA, 2 being indeterminate, and 3 being INV. We calculated the accuracy of the radiologists’ scores and further compared our radiomics model, M_R_, with the results from the radiologists (M_HR_). Finally, we integrated the probability obtained from the radiomics model, M_R_, with the radiologists scoring (1 to 3) to obtain the combined human and machine-based interpretations (M_R+HR_).

### Statistical Analysis

Statistical analysis was performed using MATLAB 2015 and R. version 3.5.3. A two-sided p-value (<0.05) was considered significant for all the statistical analyses.

Looking at the radiomic feature pool, radiomic feature stability and reproducibility were evaluated using the RIDER test-retest dataset (27). This dataset contains 31 lung cancer patients - scanned two times, 15 min apart. These scans were used for calculating the intraclass correlation coefficient (ICC) for each feature vector, which measures the similarity between two feature vectors. Considering the threshold of 0.85, all feature vectors having a value less than this threshold were removed from the analysis.

Within an unsupervised clustering analysis, hierarchical clustering and principal component analysis (PCA) combined with K-means clustering was performed on D_Train_. The clustering results were compared against ground truth for calculating the clustering accuracy.

For feature selection and building a classifier, 300 iterations of threefold cross-validation were performed within the training dataset, D_Train_. The minimum redundancy maximum relevance (mRMR) feature selection algorithm (28) was implemented within the cross-validation setting to select the top-performing radiomic features that discriminate INV from MIA/AIS. MRMR identifies a set of features that maximally distinguished two classes while minimizing intra-feature correlation. A maximum of five features was selected to prevent overfitting due to the curse of dimensionality arising from an overabundance of features relative to the sample size. mRMR was performed using MATLAB software with a feature selection toolbox for C. The top radiomic feature set was further analyzed using box-and-whisker plots and qualitative feature maps comparing feature expressions between MIA/AIS and invasive adenocarcinomas.

**Figure 1:**
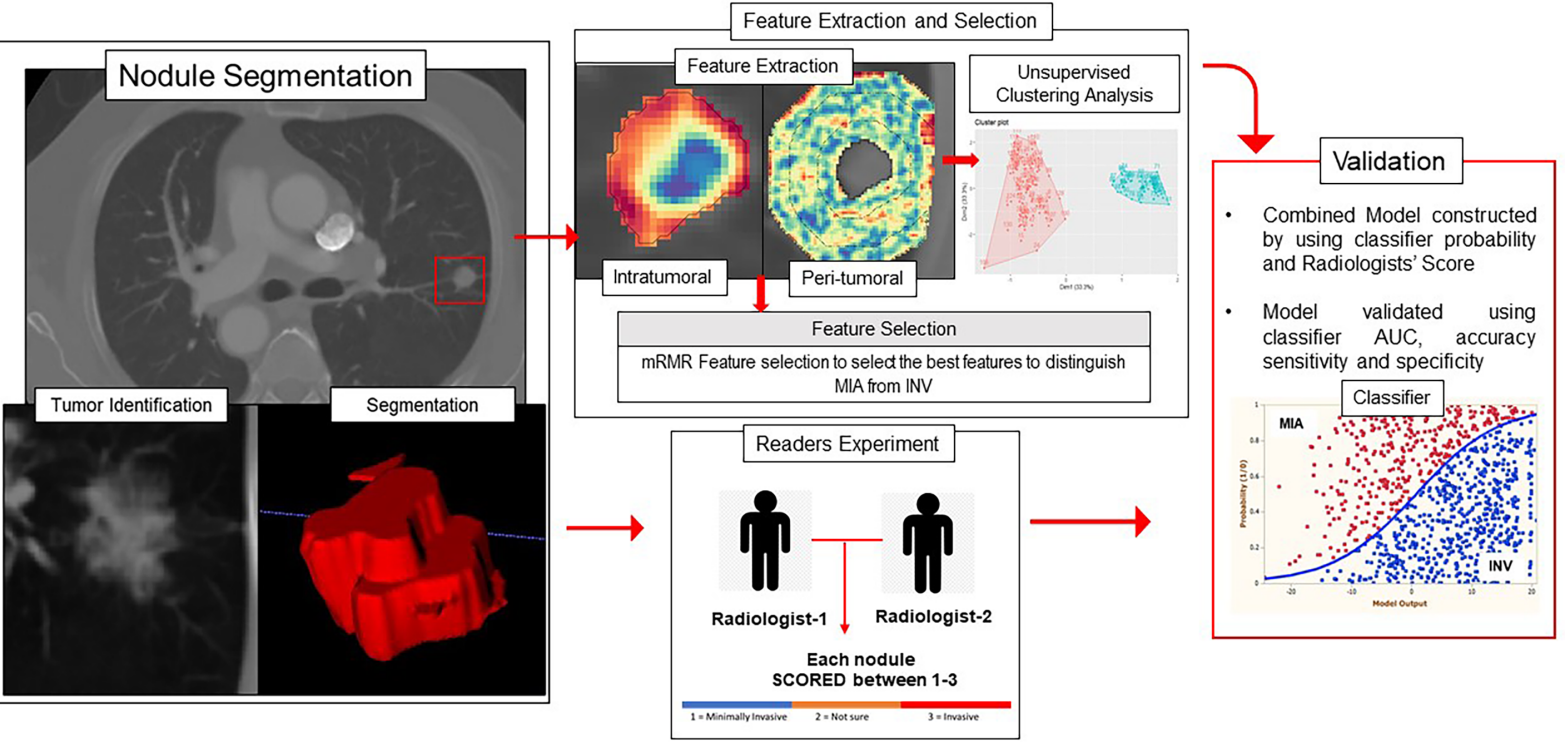
Overall workflow diagram. The nodules were segmented on the CT scans, and intratumoral and peritumoral features were extracted using MATLAB 2015. The top features were selected using the mRMR feature selection method. The validation of the radiomics model was performed using unsupervised clustering and supervised classification-based approaches.

To evaluate classifier performance, the area under the receiver operating curve (AUC), accuracy, sensitivity, and specificity were calculated for training and validation datasets. The significance of the addition of a nodule area to the radiomic model was calculated using DeLong’s test and the corresponding p-value (29). **Figure 1** shows the overall pipeline of the procedure.

## Results

### Baseline Characteristics

**Figure 2:**
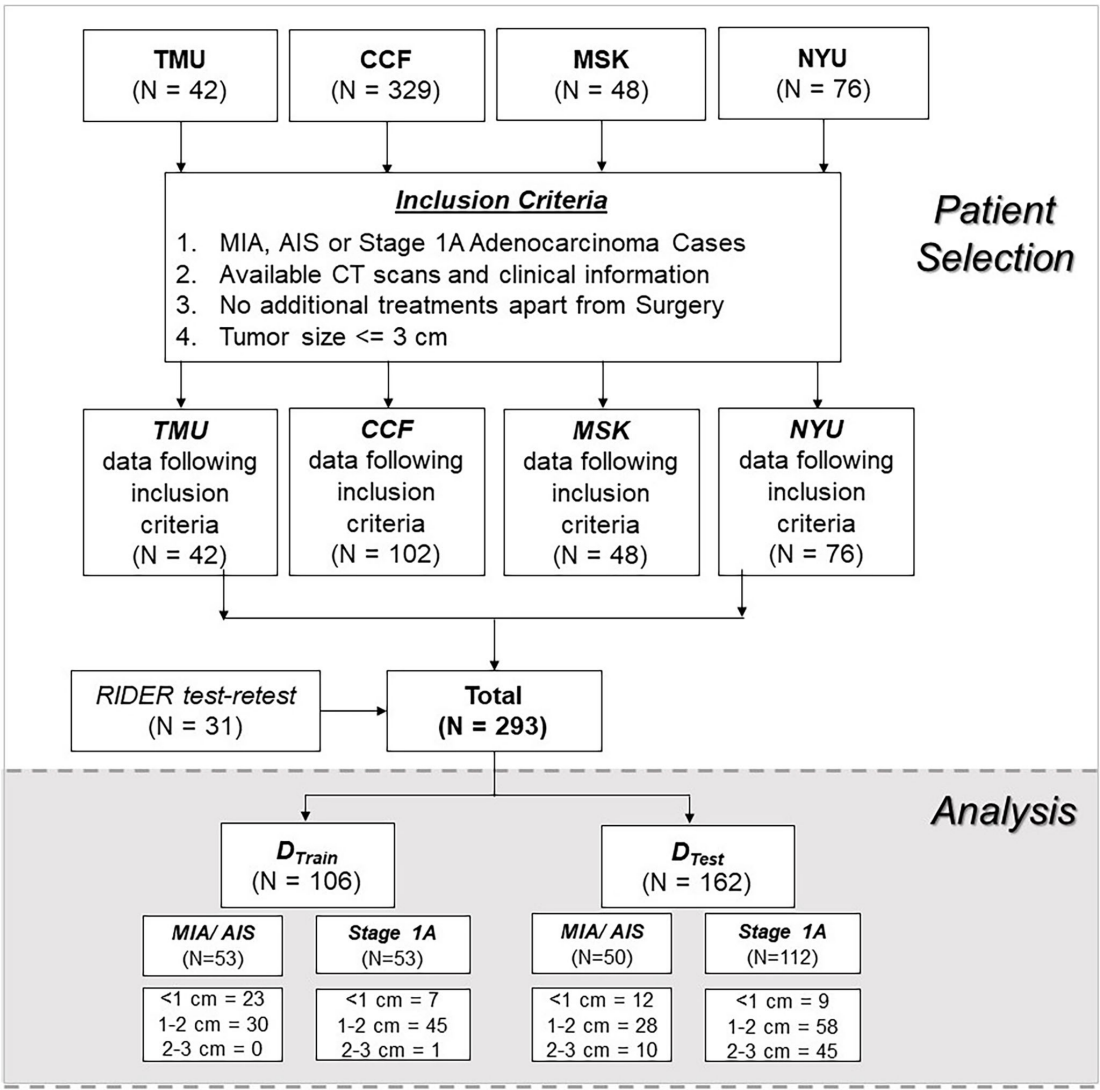
Data source and CONSORT diagram for patient selection.

Of the 268 nodules, 103 nodules were pathologically confirmed as pre-invasive lesions (AIS, n = 2) and minimally invasive lesions (MIA, n = 101), whereas 165 were confirmed as invasive lesions (INV = 165). **Figure 2** shows the datasets and patient inclusion criteria along with training and testing set distributions.

**Figure 3:**
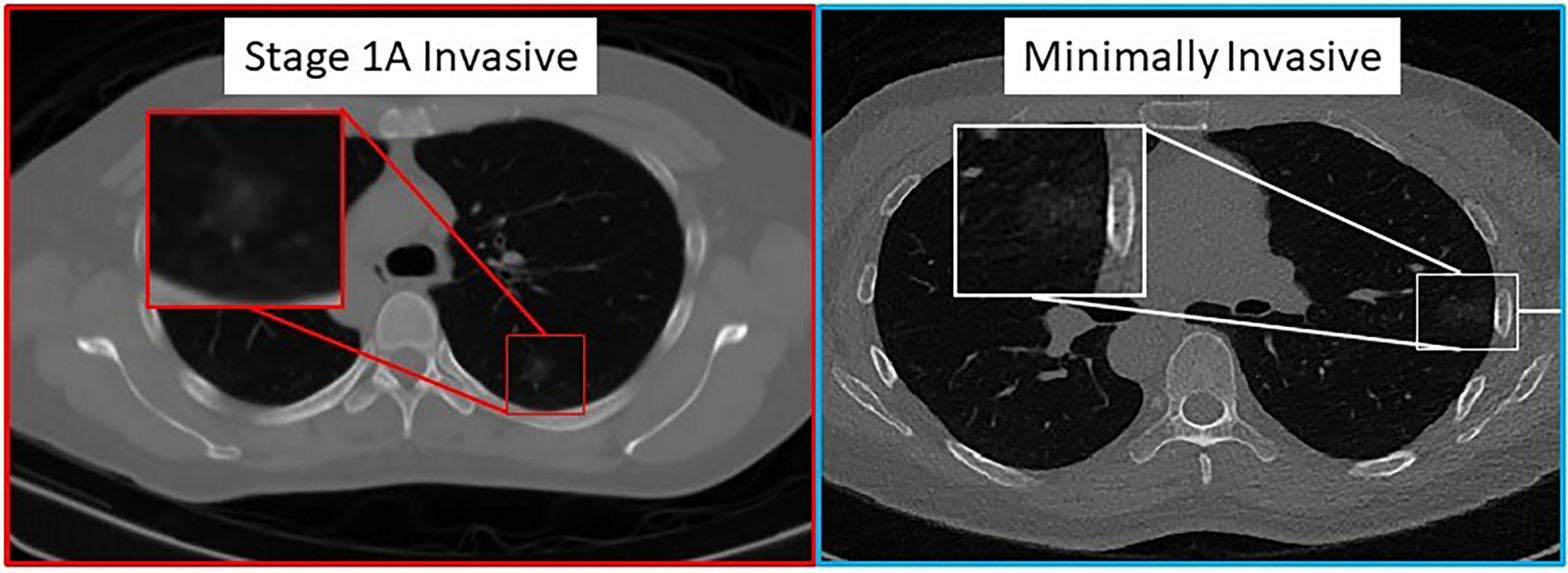
Pathologically proven INV (left image) and MIA (right image) cases presenting as predominantly ground-glass nodular densities which are indistinguishable on CT imaging.

**Figure 3** shows an example of CT scans with INV and MIA lesions.

### Experiment 1 – Differentiating Minimally Invasive Adenocarcinoma From Invasive Adenocarcinoma

#### Unsupervised Clustering

The extracted radiomic feature pool, that is, the combination of intratumoral textural and peritumoral textural radiomics features, was used within the principal component analysis (PCA) and k-means clustering to perform unsupervised clustering analysis. The optimal number of clusters was two using the first three principal components on D_Train_. The constructed clusters had an accuracy of 73.1%. The compactness within the clusters, that is, how similar the members within the same group are, was 62.8%. The validation of the constructed cluster was performed using the silhouette coefficient (silhouette width). The silhouette plot (30) suggests that the clustering using the two groups was optimal with no negative silhouette width and most cluster values > 0.5 (**Appendix 1**).

**Figure 4:**
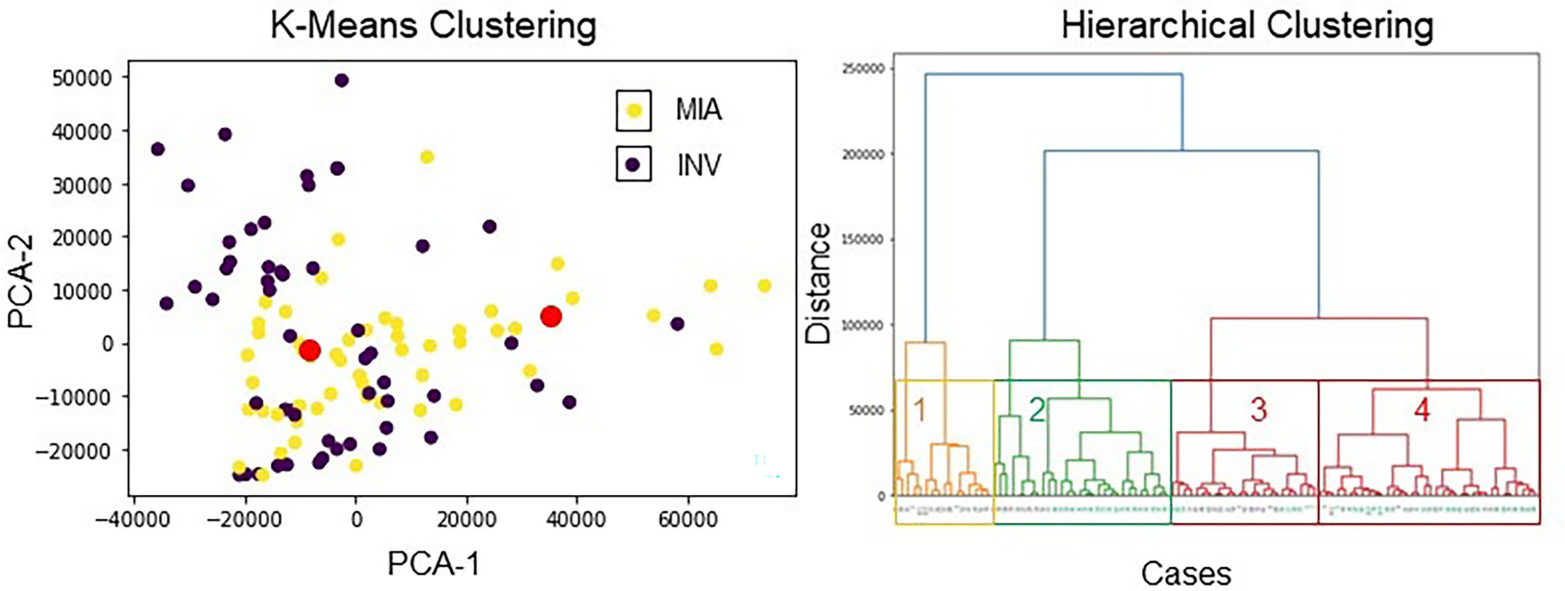
Unsupervised clustering analysis using radiomic features. (left image) K-means clustering with 4 clusters. The red dots show the centroids of the three clusters obtained *via* K-means clustering. The violet points represent INV patients, and the yellow points depict MIA patients. The two distinct clusters had an accuracy of 73.13% to distinguish MIA from INV cases. (right image) Hierarchical clustering using all features. On the x-axis, black color stands for the INV cases, and aquamarine color stands for the MIA cases.

Using the entire extracted radiomic feature pool, within the hierarchical clustering analysis, we observed the 4 obvious clusters of patients. Cluster 1 and Cluster 3 were associated with INV cases (cluster 1 = 100%, cluster 3 = 62.5% INV cases), whereas clusters 2 and 4 were associated with MIA cases (cluster 2 = 71.4%, cluster 4 = 75% MIA cases). The results of unsupervised clustering analysis are shown in **Figure 4**.

The unsupervised clustering analysis suggests that the majority of INV adenocarcinoma cases were clustered together, and MIA/AIS patients were clustered together. Collectively, these results suggest that these specific patient groups have distinct radiomic signatures.

#### Supervised Analysis and Selecting the Top Differentiating Features

**Figure 5:**
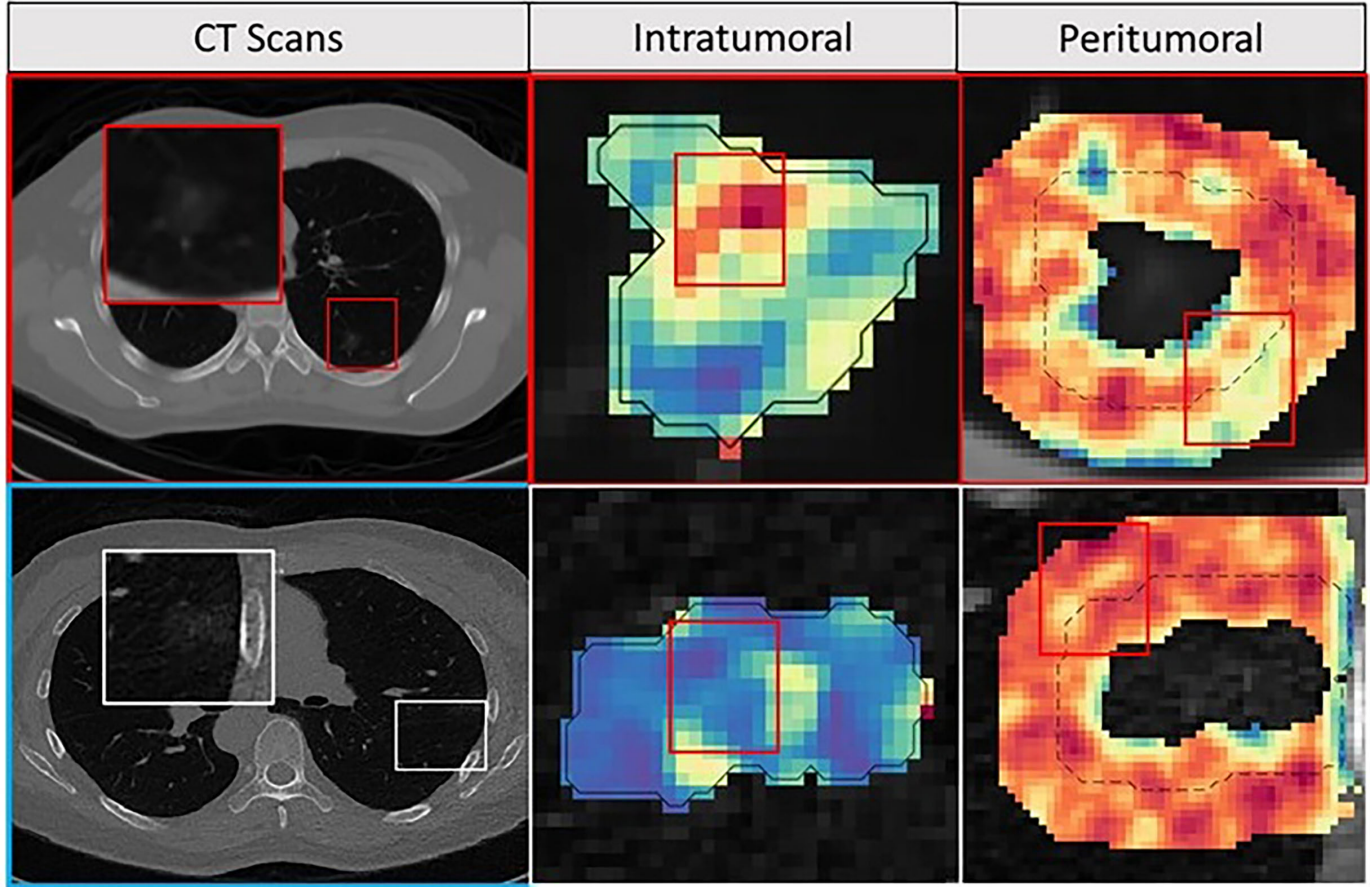
Feature maps. The first row depicts INV patient, and the bottom row depicts an MIA patient with an axial CT image as well as corresponding peritumoral and intratumoral feature maps. For the INV case, the feature maps had a higher feature expression compared to the MIA cases suggesting association between chaotic/disturbed microarchitecture and tumor invasiveness.

During feature discovery for the model M_R_ within D_Train_, the top 5 features identified included a peritumoral (CoLlAGe feature family) and 4 intratumoral features (Laws, Laplace, and Haralick feature family). The details of the top selected five features, along with their boxplots, are illustrated in **Appendix 1**. INV cases were observed to have a higher expression of intratumoral features compared to MIA cases. **Figure 5** shows the feature expression maps for the INV and MIA cases. The notations of various models constructed using these features are explained in **Table 1**.

**Table 1:** Model notations.

Model	Notations
Radiomics Model	**M_R_**
Clinical Model	**M_A_**
Radiomics-Clinical Model	**M_R+A_**
Human Reader Model	**M_HR_**
Integrated Human Reader and Radiomics Model	**M_HR+R_**

On the training cohort (D_Train,_ N=106), the logistic regression AUC for M_R_ was 0.917 [0.87-0.97]. The same classifier, within an independent blinded test set (D_Test,_ N=162), M_R_ yielded an AUC of 0.88 (**Table 2**).

**Table 2:** AUC comparison for logistic regression model trained with tumor area M_A_, radiomic features, M_R_ and combined radiomic and area based models, M_R+A_. P-value is calculated to observe the added benefit of tumor area in the radiomics model, M_R_.

		# of cases	Area M_A_	Radiomics M_R_	Rad + area M_R+A_	P (wrt area)
**Training**			0.73 [0.64-0.83]	0.917 [0.87-0.97]	0.95 [0.916-0.987]	3.013e-06
**Testing**	**All**		0.665	0.862	0.869	1.362e-05
	**0-1 cm**	22	0.79	0.759	0.713	0.492
	**1-2 cm**	87	0.61	0.919	0.926	1.057e-05
	**2-3 cm**	45	0.57	0.954	0.836	2.136e-05

Next, within the subgroup analysis, we noticed the radiomic model, M_R_, was consistent in distinguishing INV from MIA. Further, M_R_ is largely unaffected by the size of the nodule (**Table 2**).

Further, when the area of the nodule was integrated within the logistic regression classifier along with the radiomic features, M_R+A_, there was no statistically significant improvement in AUC on the validation set as compared to M_R_ standalone.

### Experiment 2 – Comparing the Radiomics Analysis With Readers

We performed the analysis with individual radiologists (M_HR_) along with the combined performance with the classifier (M_R + HR_). Reader 1 had an AUC of 0.815 and an accuracy of 0.748 for predicting MIA cases from INV cases, whereas Reader 2 had AUC and accuracy of 0.796 and 0.742, respectively (**Table 3**).

**Table 3:** AUC comparison for logistic regression model trained with radiologists’ interpretations, M_HR.,_ radiomic features, M_R,_ and combined radiomic and area-based models, M_R+HR_ on the test set. P-value is calculated to observe the added benefit of M_HR_ in the radiomics model, M_R_.

		Radiologist 1 M_HR1_	Radiologist 2 M_HR2_	Classifier M_R_	Combined M_R+HR_	P-Value	
						M_R_	M_HR1_
D_Test_ **Entire dataset**	AUC	0.815	0.796	0.861	**0.909**	0.041	4.1 e(-5)
	Accuracy	0.748	0.742	0.788	0.828		
	Sensitivity	0.800	0.792	0.820	0.760		
	Specificity	0.723	0.640	0.772	0.861		
D_Test_ **0-1 cm**	AUC	0.528	0.505	0.759	**0.796**	0.289	0.031
	Accuracy	0.571	0.571	0.667	0.761		
	Sensitivity	0.833	1.00	0.583	0.750		
	Specificity	0.223	0.00	0.778	0.778		
D_Test_ **1-2 cm**	AUC	0.831	0.794	0.916	**0.928**	0.267	0.0015
	Accuracy	0.771	0.747	0.843	0.892		
	Sensitivity	0.857	0.818	0.893	0.893		
	Specificity	0.727	0.607	0.818	0.891		
D_Test_ **2-3 cm**	AUC	0.856	0.898	0.953	**0.963**	0.624	0.025
	Accuracy	0.800	0.844	0.756	0.911		
	Sensitivity	0.861	0.889	1	0.945		
	Specificity	0.556	0.667	0.694	0.778		

Bold numbers represent the AUCs.

Within nodules <1 cm size, the classifier demonstrated an improvement over the radiologists’ interpretations.

Finally, we combined the classifier predictions with the radiologists’ scores and constructed the combined model M_R+HR_. M_R+HR_ achieved an average AUC of 0.909 on D_Test,_ corresponding to the highest AUC among all models (M_R_= 0.861, p=0.041; M_HR1 =_ 0.815, p <0.001; M_HR2 =_ 0.796, p<0.001).

## Discussion

Current CT technologies have improved and expedited early lung nodule diagnosis. Patients diagnosed as MIA survive well, postoperative recurrence and lymph node metastasis are rare, and 5-year survival rate is close to 100%. In contrast, patients with INV have reduced five-year survival (31–33). Lobectomy is considered the standard surgical treatment for INV patients (13). Prior studies using CT scans features of air bronchograms and borders have not been able to accurately distinguish invasive lesions (32). An accurate way to determine the lesion’s invasiveness pre-operatively on routine chest CT scans would be beneficial in guiding the need for the timing of resection and potentially amount of resection (13).

In our work, we developed a computerized model using textural patterns known as radiomics to accurately differentiate MIA from INV cases from pre-treatment baseline CT scans from four different institutions. We observed that radiomic features extracted from intra- and peritumoral regions of these lung nodules harbor information related to nuances of the tissue properties not apparent to the naked eye. Additionally, in our analysis, two radiologists examined these scans in a blinded fashion. They scored them visually, and the integration of radiologists’ interpretation with the classifier performance yielded the highest diagnostic accuracy on the test set (AUC = 0.909).

Although there have been previous successful attempts to examine GGOs *via* radiomics analysis (20, 21, 34, 35), most studies focus on textural patterns extracted from within the lung lesions to differentiate MIA from INV lesions. Specifically, most of them employed features focused on the gray level co-occurrence-based matrix and wavelet-based feature families for identifying INV cases (20, 36). A few studies have further integrated clinical and morphological features into the radiomics model to improve model accuracy (20, 37).

Two of the top five features identified by our radiomics based supervised approach corresponded to the gray-level co-occurrence-based feature (GLCM) families which is in line with previously published results (20). In addition, we also noticed Laws and Laplace features extracted from within the nodule to be among the top set of discriminating features. These two feature families (Laws and Laplace) examine higher-order frequency content of the given region of interest (25). We noticed a higher expression of all intratumoral features for INV when compared to MIA nodules. The elevated expressions of these radiomic features could reflect more chaotic and haphazard microarchitecture within the comparatively high-risk invasive tumors (**Figure 5**).

In our work, we also interrogated the tumor environment (TME) surrounding the nodule (i.e., peritumoral region) to evaluate its utility in providing complementary information with respect to disease diagnosis. We defined the radiomic profile of these GGO nodules during the feature discovery portion using a combination of intra- and peritumoral regions. Within our analysis, we noticed one of the top five features was from the peritumoral region. The feature was observed from within the 3 to 6 mm region outside the nodule. Recent studies have shed new light on this complex interaction between tumor and host immune cells and immune responses. In work by Altorki et al. (22), the authors demonstrated the role of TME for progression for pre-invasive to invasive adenocarcinoma lesions. They observed a dominant regulatory T cell-mediated immune suppression initiated at the precursor level sustained with rising intensity throughout malignant progression. Few studies also show that these perinodular radiomic features may reflect tumor microarchitecture changes or be capturing the presence of tumor-infiltrating lymphocytes (TILs) (18). We noticed an increased peritumoral CoLlAGe feature (24) expression for MIA cases.

Specifically with respect to the perinodular region, in work by Wu G. et al. (36), the authors did not observe an improvement in AUC with the addition of radiomic features from the perinodular region to differentiate INV cases from MIA and AIS (p = 0.11). They observed the most predictive features to emanate from the ground-glass and solid regions of the nodule. Whereas in the work by Wu L. et. al (38)., the authors show the utility of perinodular features for the same clinical problem. However, in our analysis, we noticed CoLlAGe peritumoral radiomic features to be statistically significant between the training and testing cohorts (**Appendix 1**; <0.01). CoLIAGe captures higher-order co-occurrence patterns of local gradient tensors at a voxel level and has been shown to be diagnostic and prognostic for a variety of disease indications (17, 18, 24). Additionally, in our analysis, we included the complete GGOs in addition to semisolid nodules unlike in the study by Wu et al. (36).

We further evaluated and compared our radiomic model with the tumor diameter. Studies show the two-dimensional diameter of the nodule to be one of the strongest predictors for pulmonary nodule risk classification in the quantitative CT image analysis. In work by Xu et al. (34), the authors noticed the diameter of GGOs to be significantly different in MIA and INV nodules, and a conventional model constructed using clinical and quantitative features (such as age, diameter, and density) yielded the best AUC (0.848; 95% CI = 0.750-0.946). The authors observed that the addition of radiomic features to the clinical and quantitative models did not improve the performance of the combined model (34). In contrast, multiple studies have reported the added benefit of radiomics to clinical and quantitative models (20, 37). In a study by Weng et al. (20), the authors constructed a nomogram using lesion shape, solid component, and radiomics features from the nodule to obtain an AUC of 0.88. Similarly, Luo et al. (37) used three CT features (pleural indentation, solid component size, and solid component proportion) and one radiomic feature to help differentiate invasive pulmonary adenocarcinoma (IPA) from non-IPA to achieve a final AUC of 0.903. Interestingly, in our analysis, the radiomic model was superior to the model constructed with the nodule area in both training and testing sets. The addition of the nodule diameter to the radiomics model did not improve the performance especially in the independent validation set (D_Train_:0.95 [0.92-0.98] from 0.92 [0.87-0.97], p=0.03; D_Test_:0.869 [0.80-0.93] from 0.862 [0.79-0.93], p=0.86) even though individual tumor diameter was statistically significant in differentiating MIA and INV nodules (**Appendix 1** D_Train_< 0.05; D_Test_< 0.05). We further created a subset of nodules with a diameter of less than 10 mm. We noticed that our radiomics classifier was prognostic even within the smaller nodules, giving an AUC of 0.76 [0.53-0.98] on these smaller lesions.

Another unique aspect of our study included integrated classifier performance with expert radiologists’ visual assessment of the tumors. We noticed that the classifier had an overall improvement of ~4.5% compared to the radiologists’ interpretations. We noticed that the radiologists had high sensitivity, but poor specificity. After combining the probabilities of the machine learning classifier with the radiologists’ score, the model AUC improved to 0.909 from 0.867 of the classifier model (p<0.05) and 0.816 of the radiologists’ model (p<0.05).

Overall, our study has three main novel contributions including the multi-institutional nature, the addition of novel radiomics descriptor in the analysis, and human-machine comparison and integration to create consensus and accurate models.

Despite the progress made in this study, our work has some limitations. First, the developed model is completely retrospective in nature. For a successful transition into the clinically deployable model, a prospective evaluation will be required. Second, even though the analysis had multiple institutions, we did not truly validate the model independently since all the cases from individual sites were collapsed and subsequently randomly divided into training and testing sets. Future work will entail prospective data as well as validation on data from sites independent from those employed for developing the model.

## Data Availability Statement

The original contributions presented in the study are included in the article/**Supplementary Material**. Further inquiries can be directed to the corresponding author.

## Author Contributions

PV, DJ, MB, HP, RG, FJ, KL-CH, G-YL, and VV were involved in collecting data. PV and KB performed the analysis and wrote the first draft of the manuscript. AG and PR performed the radiologists’ evaluation which was integrated with the imaging model. AM and PL outlined the experimental design. All the authors had access to data and approved the manuscript. AM decided to submit the manuscript. All authors contributed to the article and approved the submitted version.

## Funding

Research reported in this article was supported by The National Cancer Institutes, USA under award numbers 1U24CA199374-01, R01CA249992-01A1, R01CA202752-01A1, R01CA208236-01A1, R01CA216579-01A1, R01CA220581-01A1, R01CA257612-01A1, 1U01CA239055-01, 1U01CA248226-01, 1U54CA254566-01, 2P50CA150964-06A1, and R01CA196643-01; The National Heart, Lung, and Blood Institute, 1R01HL15127701A1; The National Institute of Biomedical Imaging and Bioengineering, 1R43EB028736-01; The National Center for Research Resources under award number 1 C06 RR12463-01; VA Merit Review Award IBX004121A from the United States Department of Veterans Affairs Biomedical Laboratory Research and Development Service; the Office of the Assistant Secretary of Defense for Health Affairs, through the Breast Cancer Research Program (W81XWH-19-1-0668), the Prostate Cancer Research Program (W81XWH-15-1-0558, W81XWH-20-1-0851), the Lung Cancer Research Program (W81XWH-18-1-0440, W81XWH-20-1-0595), and the Peer Reviewed Cancer Research Program (W81XWH-18-1-0404); the Kidney Precision Medicine Project (KPMP) Glue Grant; the Ohio Third Frontier Technology Validation Fund; and the Clinical and Translational Science Collaborative of Cleveland (UL1TR0002548) from the National Center for Advancing Translational Sciences (NCATS) component of the National Institutes of Health and NIH roadmap for Medical Research. We also acknowledge The Wallace H. Coulter Foundation Program in the Department of Biomedical Engineering at Case Western Reserve University and sponsored research agreements from Bristol Myers Squibb, Boehringer-Ingelheim, and AstraZeneca. The content is solely the responsibility of the authors and does not necessarily represent the official views of the National Institutes of Health, the US Department of Veterans Affairs, the Department of Defense, or the United States Government. The funders were not involved in the study design, collection, analysis, interpretation of data, the writing of this article or the decision to submit it for publication.

## Author Disclaimer

The content is solely the responsibility of the authors and does not necessarily represent the official views of the National Institutes of Health, the U.S. Department of Veterans Affairs, the Department of Defense, or the United States Government.

## Conclusion

Intratumoral and peritumoral radiomic features show the ability to distinguish INV from MIA and AIS. With additional prospective validation, this radiologic tool could assist in clinical decision-making as to when tumoral pulmonary lesions should be treated and could also be useful in helping guide the extent of resection.

## Conflict of Interest

The authors declare that the research was conducted in the absence of any commercial or financial relationships that could be construed as a potential conflict of interest.

## Publisher’s Note

All claims expressed in this article are solely those of the authors and do not necessarily represent those of their affiliated organizations, or those of the publisher, the editors and the reviewers. Any product that may be evaluated in this article, or claim that may be made by its manufacturer, is not guaranteed or endorsed by the publisher.

## Abbreviations

NSCLC, non-small cell lung cancer; MIA, minimally invasive adenocarcinoma; AIS, adenocarcinoma in situ; INV, invasive; GGO, ground-glass opacities; ROI, region of interest; CT, computed tomography; BAC, broncho-alveolar carcinoma; AUC, area under the receiver operating characteristic curve; mRMR, minimum redundancy maximum relevance; ICC, intraclass correlation; M_R_, radiomics model; M_A_, clinical model; M_R+A_, combined radiomics-clinical model; M_HR_, human reader model; M_R+HR_, integrated radiologist and radiomics model; CoLlAGe, co-occurrence of local anisotropic gradient.
