# Supplementary material for: Combined Radiomic and Visual Assessment for Improved Detection of Lung Adenocarcinoma Invasiveness on Computed Tomography Scans: A Multi-Institutional Study
Source: Front Oncol. 2022 May 30;12:902056. doi: 10.3389/fonc.2022.902056 (PMC9190758; doi:10.3389/fonc.2022.902056)
Supplement: Supplementary file 1 [file DataSheet_1.docx]

**Appendix-1**

**Section 1: Radiomic Feature Extraction.**

Lung nodules were annotated by an expert radiologist using 3D-slicer (3D Slicer, version 4.6: NIH-funded; <https://www.slicer.org>) and a freehand tool. Intratumoral and peritumoral areas were selected using these annotations. For intratumoral region, the annotated tumor is used as the area of interest. Following annotations, the peritumoral region corresponding to the lesion was defined using the following steps. Firstly, a morphological operation of dilation was performed to capture the area outside the nodule, up to a radial distance of 15mm from the lesion boundary. The choice of peri-nodular size was based on previous findings, where a resection margin >15mm did not have a prognostic effect in the context of disease recurrence. The intranodular mask was then subtracted from this dilated mask to obtain a ring of lung parenchyma immediately around the nodule. The perinodular region was subsequently divided into five equally spaced 3-mm rings.

Radiomic features were extracted from the three slices having the maximum area of the tumor using software developed in the Center of Computational Imaging and Personalized Diagnostics, Case Western Reserve University, implemented on a MATLAB release 2016a platform (MathWorks, Natick, MA, USA).

A total of 124 textural descriptors were commutated voxel-wise across the three slices from each of the annotated region. The features included 48 from Gabor feature family, 13 from Haralick, 13 from Collage, 25 from Laws, and 25 from Laplace feature families. The First-order statistics (mean, median, SD, skewness, and kurtosis) were calculated from each feature vector. Table-S1 explains all the extracted features.

**Section 2: Total Extracted Radiomic Features.**

The Table-S1 explains all the extracted features.

**Table S.1** – Total Radiomic Features extracted from intratumoral and annular ring-shaped peritumoral regions. A total of 6 statistics- (mean, median, kurtosis, skewness, standard deviation, and range) were calculated for each feature.

| **Radiomic Feature Family** | **Radiomic Feature Parameters** | **Radiomic Feature Family** | **Radiomic Feature Parameters** |
| --- | --- | --- | --- |
| **Haralick Feature Family** | Entropy, Window Size = 5 | **Laws Energy Feature Family** | Level, Level |
|  | Energy, Window Size = 5 |  | Level, Edge |
|  | Inertia, Window Size = 5 |  | Level, Spot |
|  | Inverse Difference Moment, Window Size = 5 |  | Level, Wave |
|  | Correlation, Window Size = 5 |  | Level, Ripple |
|  | Information Measure of Correlation-1, Window Size = 5 |  | Edge, Level |
|  | Information Measure of Correlation-2, Window Size = 5 |  | Edge, Edge |
|  | Sum Average, Window Size = 5 |  | Edge, Spot |
|  | Sum Variance, Window Size = 5 |  | Edge, Wave |
|  | Sum Entropy, Window Size = 5 |  | Edge, Ripple |
|  | Difference Average, Window Size = 5 |  | Spot, Level |
|  | Difference Variance, Window Size = 5 |  | Spot, Edge |
|  | Difference Entropy, Window Size = 5 |  | Spot, Spot |
| **Gabor Wavelet Feature Family** | Orientation=0, Bandwidth=1, Frequency=0 |  | Spot, Wave |
|  | Orientation=0, Bandwidth=1, Frequency=2 |  | Spot, Ripple |
|  | Orientation=0, Bandwidth=1, Frequency=4 |  | Wave, Level |
|  | Orientation=0, Bandwidth=1, Frequency=6 |  | Wave, Edge |
|  | Orientation=0, Bandwidth=1, Frequency=8 |  | Wave, Spot |
|  | Orientation=0, Bandwidth=1, Frequency=10 |  | Wave, Wave |
|  | Orientation=π/8, Bandwidth=1, Frequency=0 |  | Wave, Ripple |
|  | Orientation=π/8, Bandwidth=1, Frequency=2 |  | Ripple, Level |
|  | Orientation=π/8, Bandwidth=1, Frequency=4 |  | Ripple, Edge |
|  | Orientation=π/8, Bandwidth=1, Frequency=6 |  | Ripple, Spot |
|  | Orientation=π/8, Bandwidth=1, Frequency=8 |  | Ripple, Wave |
|  | Orientation=π/8, Bandwidth=1, Frequency=10 |  | Ripple, Ripple |
|  | Orientation=π/4, Bandwidth=1, Frequency=0 | **Laplace Feature Family** | Level, Level |
|  | Orientation=π/4, Bandwidth=1, Frequency=2 |  | Level, Edge |
|  | Orientation=π/4, Bandwidth=1, Frequency=4 |  | Level, Spot |
|  | Orientation=π/4, Bandwidth=1, Frequency=6 |  | Level, Wave |
|  | Orientation=π/4, Bandwidth=1, Frequency=8 |  | Level, Ripple |
|  | Orientation=π/4, Bandwidth=1, Frequency=10 |  | Edge, Level |
|  | Orientation=3π/8, Bandwidth=1, Frequency=0 |  | Edge, Edge |
|  | Orientation=3π/8, Bandwidth=1, Frequency=2 |  | Edge, Spot |
|  | Orientation=3π/8, Bandwidth=1, Frequency=4 |  | Edge, Wave |
|  | Orientation=3π/8, Bandwidth=1, Frequency=6 |  | Edge, Ripple |
|  | Orientation=3π/8, Bandwidth=1, Frequency=8 |  | Spot, Level |
|  | Orientation=3π/8, Bandwidth=1, Frequency=10 |  | Spot, Edge |
|  | Orientation=π/2, Bandwidth=1, Frequency=0 |  | Spot, Spot |
|  | Orientation=π/2, Bandwidth=1, Frequency=2 |  | Spot, Wave |
|  | Orientation=π/2, Bandwidth=1, Frequency=4 |  | Spot, Ripple |
|  | Orientation=π/2, Bandwidth=1, Frequency=6 |  | Wave, Level |
|  | Orientation=π/2, Bandwidth=1, Frequency=8 |  | Wave, Edge |
|  | Orientation=π/2, Bandwidth=1, Frequency=10 |  | Wave, Spot |
|  | Orientation=5π/8, Bandwidth=1, Frequency=0 |  | Wave, Wave |
|  | Orientation=5π/8, Bandwidth=1, Frequency=2 |  | Wave, Ripple |
|  | Orientation=5π/8, Bandwidth=1, Frequency=4 |  | Ripple, Level |
|  | Orientation=5π/8, Bandwidth=1, Frequency=6 |  | Ripple, Edge |
|  | Orientation=5π/8, Bandwidth=1, Frequency=8 |  | Ripple, Spot |
|  | Orientation=5π/8, Bandwidth=1, Frequency=10 |  | Ripple, Wave |
|  | Orientation=3π/4, Bandwidth=1, Frequency=0 |  | Ripple, Ripple |
|  | Orientation=3π/4, Bandwidth=1, Frequency=2 | **CoLIAGe Feature Family** | Entropy, Window Size = 5 |
|  | Orientation=3π/4, Bandwidth=1, Frequency=4 |  | Energy, Window Size = 5 |
|  | Orientation=3π/4, Bandwidth=1, Frequency=6 |  | Inertia, Window Size = 5 |
|  | Orientation=3π/4, Bandwidth=1, Frequency=8 |  | Inverse Difference Moment , Window Size = 5 |
|  | Orientation=3π/4, Bandwidth=1, Frequency=10 |  | Correlation, Window Size = 5 |
|  | Orientation=7π/8, Bandwidth=1, Frequency=0 |  | Information Measure of Correlation-1, Window Size = 5 |
|  | Orientation=7π/8, Bandwidth=1, Frequency=2 |  | Information Measure of Correlation-2, Window Size = 5 |
|  | Orientation=7π/8, Bandwidth=1, Frequency=4 |  | Sum Average, Window Size = 5 |
|  | Orientation=7π/8, Bandwidth=1, Frequency=6 |  | Sum Variance, Window Size = 5 |
|  | Orientation=7π/8, Bandwidth=1, Frequency=8 |  | Sum Entropy, Window Size = 5 |
|  | Orientation=7π/8, Bandwidth=1, Frequency=10 |  | Difference Average, Window Size = 5 |
|  |  |  | Difference Variance, Window Size = 5 |
|  |  |  | Difference Entropy, Window Size = 5 |

**Section 3: Radiomic Feature Stability.**

Feature stability and reproducibility were evaluated using the RIDER test-retest dataset. This dataset had 31 lung cancer patients, scanned two times with 15 mins’ difference apart. Two scans of every patient were used for calculating the intraclass correlation coefficient (ICC) for each feature vector. Considering the threshold of 0.85, the analysis was performed using all feature vectors having ICC values higher than the threshold limit. After completing the above experiment, a total of 267 features were retained from the intratumoral and peritumoral feature pool of 1410. These stable features were used for further analysis for feature selection and model building.

**Section 4: CT Parameters**

The following Table-S2 explains the CT scan parameters for four different datasets.

**Table S.2** – CT parameters for the datasets used in the analysis.

| CCF | kVp | 100/ 110 | 8 |
| --- | --- | --- | --- |
|  |  | 120 | 87 |
|  |  | 130/ 135/ 140 | 7 |
|  | Convolution Kernel | B/ B30f/ B30s/ B31f/ B35f/ B35s/ B40f/ B40s/ B41f/ B60f/ B70f | 79 |
|  |  | LUNG/ Standard/ YB | 4 |
|  |  | L/V/FC12/ FC13/ FC52/ FC86/ I31f4 | 10 |
|  | Pixel Size | <0.75 mm | 25 |
|  |  | >0.75 mm and <1 mm | 76 |
|  |  | >1 mm | 1 |
|  | Slice Thickness | <1 mm | 28 |
|  |  | >1 mm and <3 mm | 63 |
|  |  | >3 mm | 11 |
| MSK | kVp | 100/ 110 | 2 |
|  |  | 120 | 46 |
|  |  | 130/ 135/ 140 | 0 |
|  | Convolution Kernel | B/ B30f/ B30s/ B31f/ B35f/ B35s/ B40f/ B40s/ B41f/ B60f/ B70f | 5 |
|  |  | LUNG/ Standard | 39 |
|  |  | FC56/I70f2/I40f1/ I70f1 | 4 |
|  | Pixel Size | <0.75 mm | 41 |
|  |  | >0.75 mm and <1 mm | 7 |
|  |  | >1 mm | 0 |
|  | Slice Thickness | <=1 mm | 3 |
|  |  | >1 mm and <3 mm | 31 |
|  |  | >3 mm | 14 |
| TMU | kVp | 80/100/ 110 | 22 |
|  |  | 120 | 19 |
|  |  | 130 | 1 |
|  | Convolution Kernel | B/ B31f | 5 |
|  |  | LUNG/ Standard/ BONE | 20 |
|  |  | T20f/ FL03/ FC08/ T20s/ T20f/ I50f3 | 17 |
|  | Pixel Size | <0.75 mm | 28 |
|  |  | >0.75 mm and <1 mm | 3 |
|  |  | >=1 mm | 11 |
|  | Slice Thickness | <1 mm | 7 |
|  |  | >1 mm and <3 mm | 9 |
|  |  | >3 mm | 26 |
| NYU | kVp | 80/100/ 110 | 25 |
|  |  | 120 | 51 |
|  | Convolution Kernel | B30f/B40f/ B60f | 29 |
|  |  | I40f1/ Br40d2/ I70f1 | 47 |
|  | Pixel Size | <0.75 mm | 71 |
|  |  | >0.75 mm and <1 mm | 5 |
|  | Slice Thickness | <=1 mm | 1 |
|  |  | >1 mm and <3 mm | 1 |
|  |  | >3 mm | 74 |

**Section 5: Unsupervised Analysis**

The unsupervised analysis was performed using all the Radiomic Feature pool set. Supplement Figure-1 shows the results for unsupervised clustering analysis on training and testing datasets.


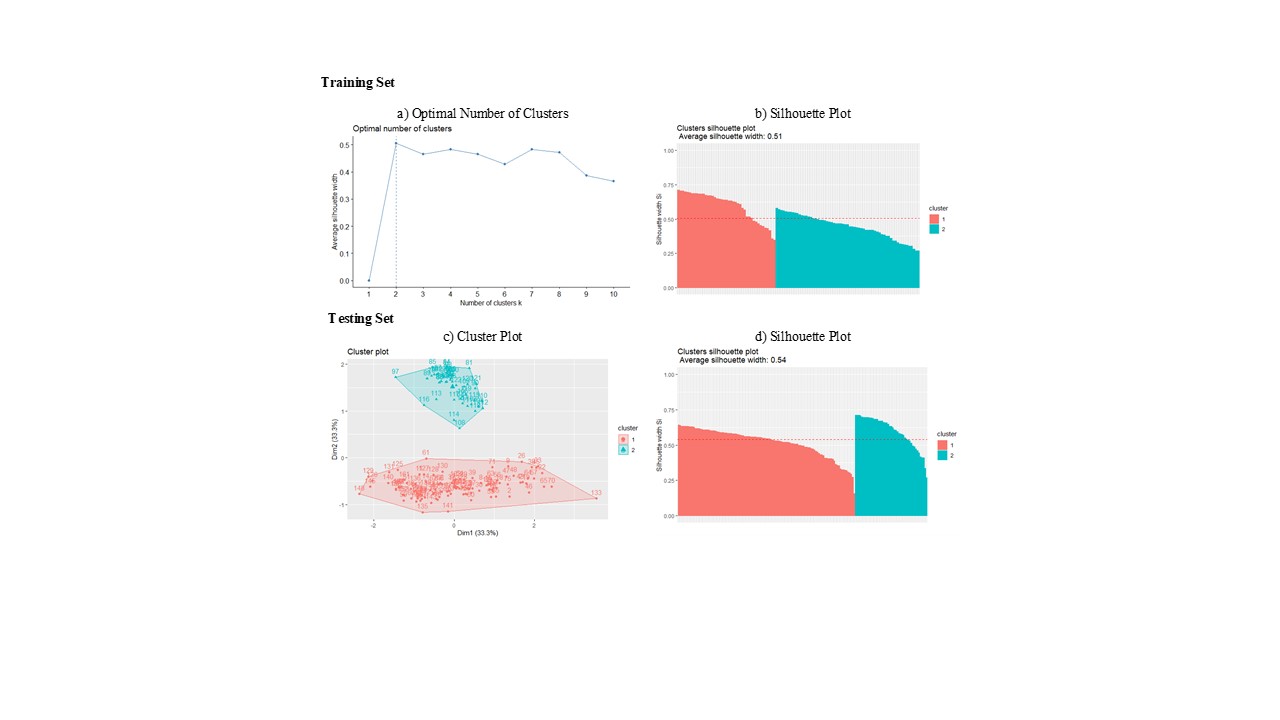


**S.Figure 1:** Unsupervised clustering results on training and testing sets. A) The selection of optimal clusters using elbow plots b) Sillhoutte plot for the training set, c) and d) clustering plots and sillhoutte plots for testing sets.

**Section 6: Boxplots of the top selected Radiomic Features**

**Top Selected Features** –

**Table S.3:** Top Selected Radiomic Features in the analysis

| **Distance** | **Feature Family** | **Statistics** |
| --- | --- | --- |
| Intratumoral | Laws- Level Spot | Range |
| Intratumoral | Haralick- Sum variance | Mean |
| Intratumoral | Laws- Level Spot | Mean |
| Intratumoral | Laplace- Spot ripples | Standard Deviation |
| Peritumoral (3-6mm) | CoLlAGe- Diff variance | Median |

Following Supplement Figures-2(a-f) shows the boxplots for the top selected features.


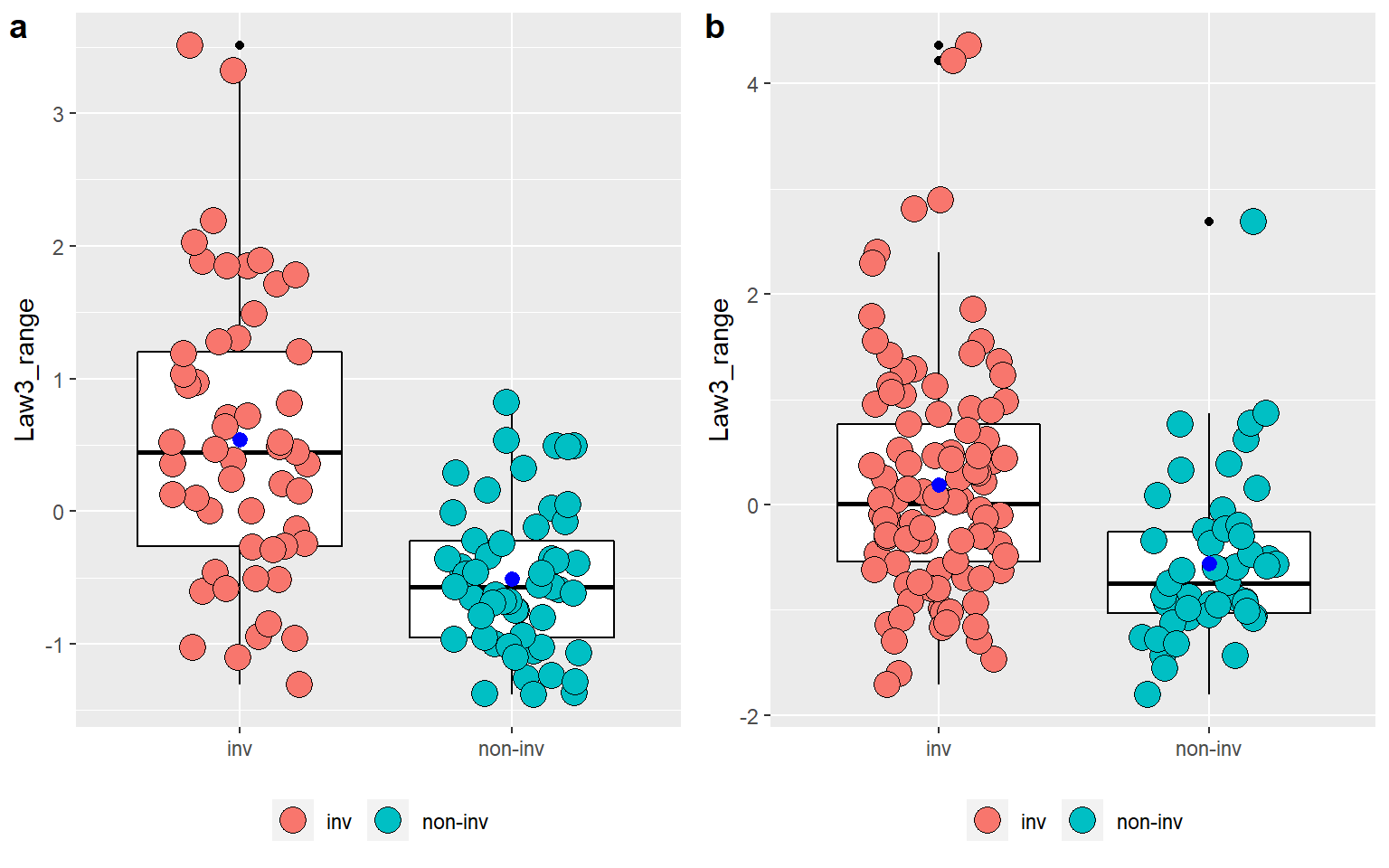


**S.Figure 2a.** Boxplot for a) training and b) testing set for intratumoral Laws-Level Spot feature


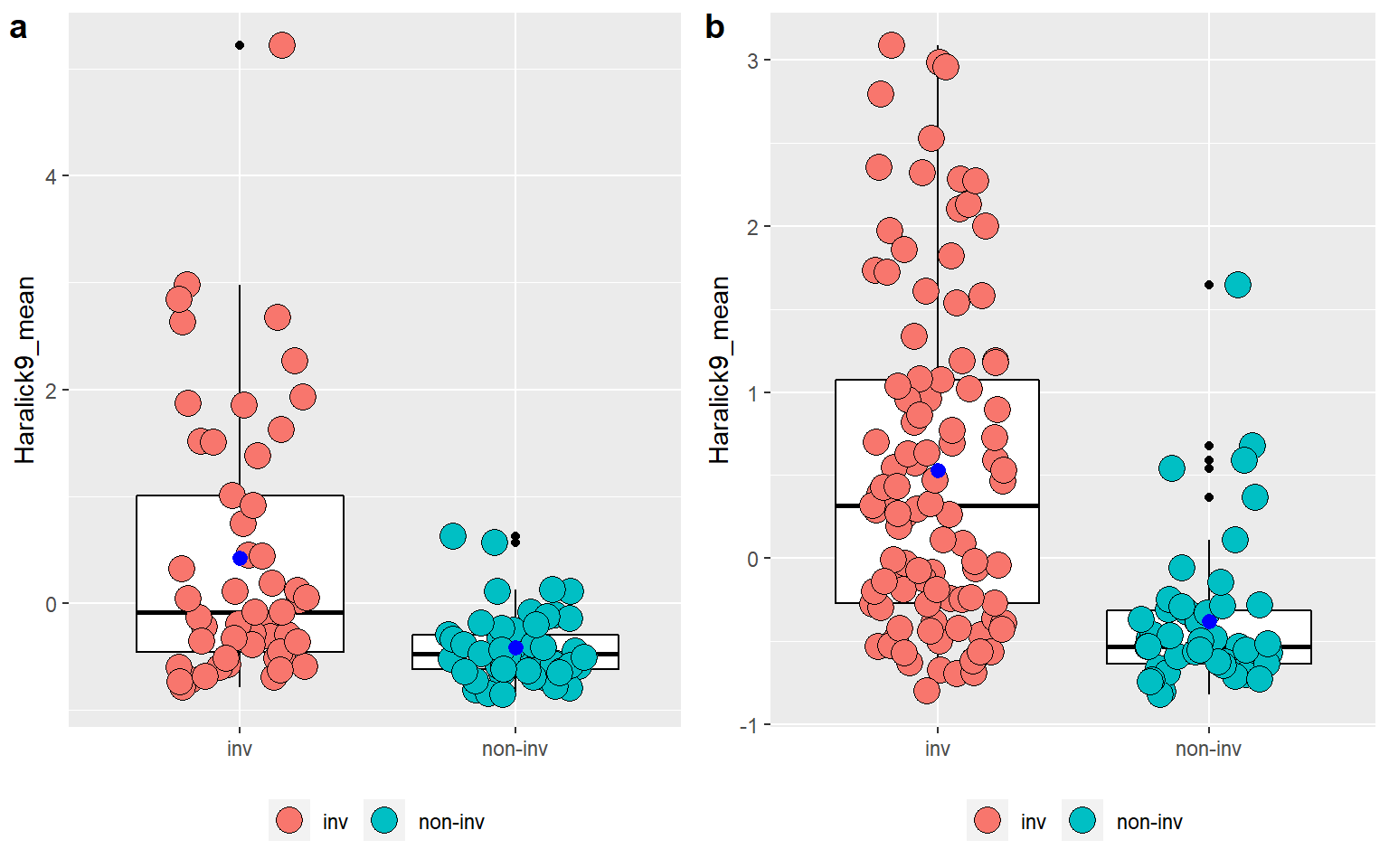


**S.Figure 2b.** Boxplot for a) training and b) testing set for intratumoral Haralick -Sum Variance feature


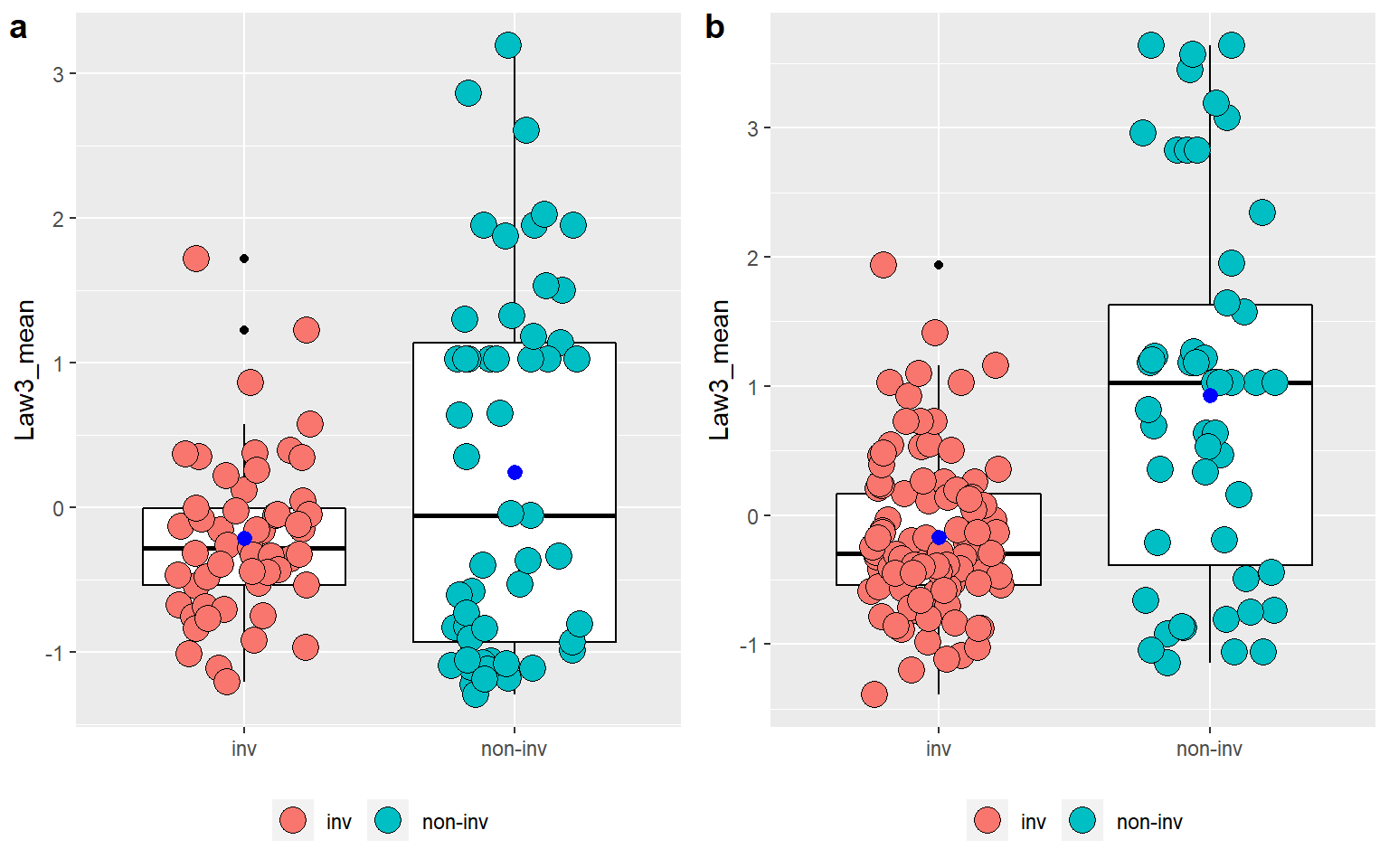


**S.Figure 2d.** Boxplot for a) training and b) testing set for intratumoral Laws -Level Spot feature


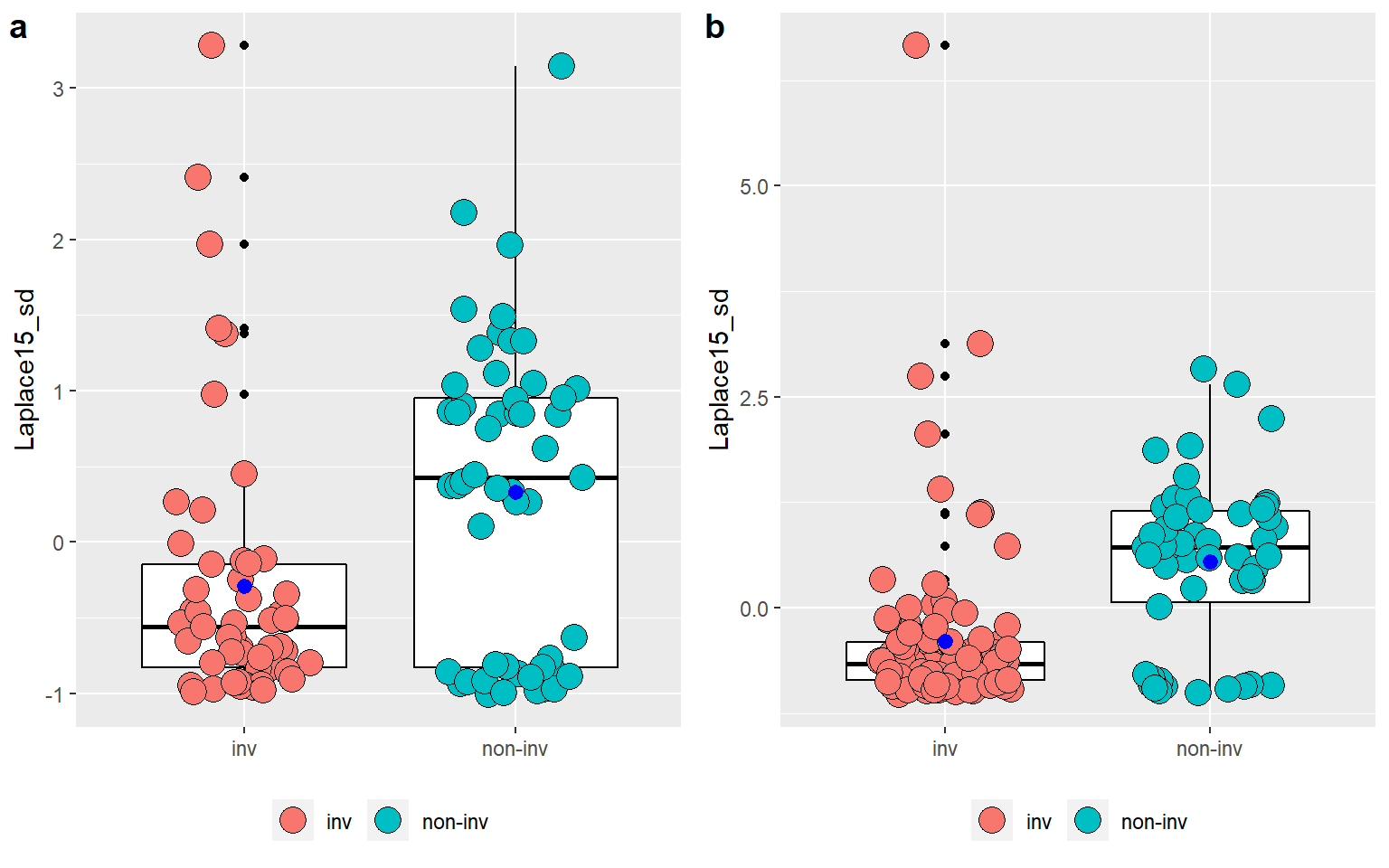


**S.Figure 2e.** Boxplot for a) training and b) testing set for intratumoral Laplace -Spot Ripple feature


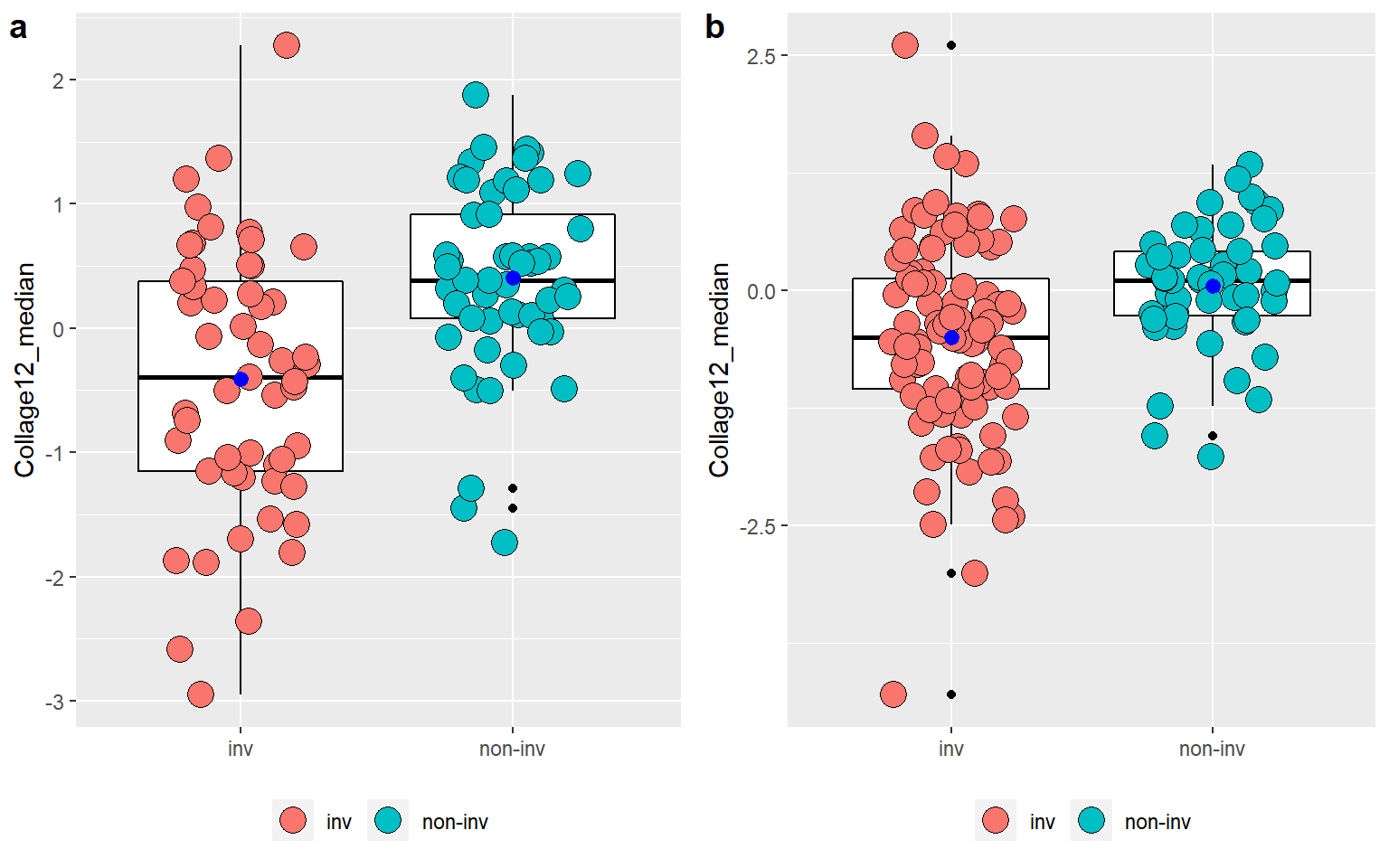


**S.Figure 2f.** Boxplot for a) training and b) testing set for peritumoral (3-6mm) Collage -difference variance feature

**Section 7: Boxplots with respect to Area**

Supplement Figure-3 shows the boxplots and for the area of the nodule.


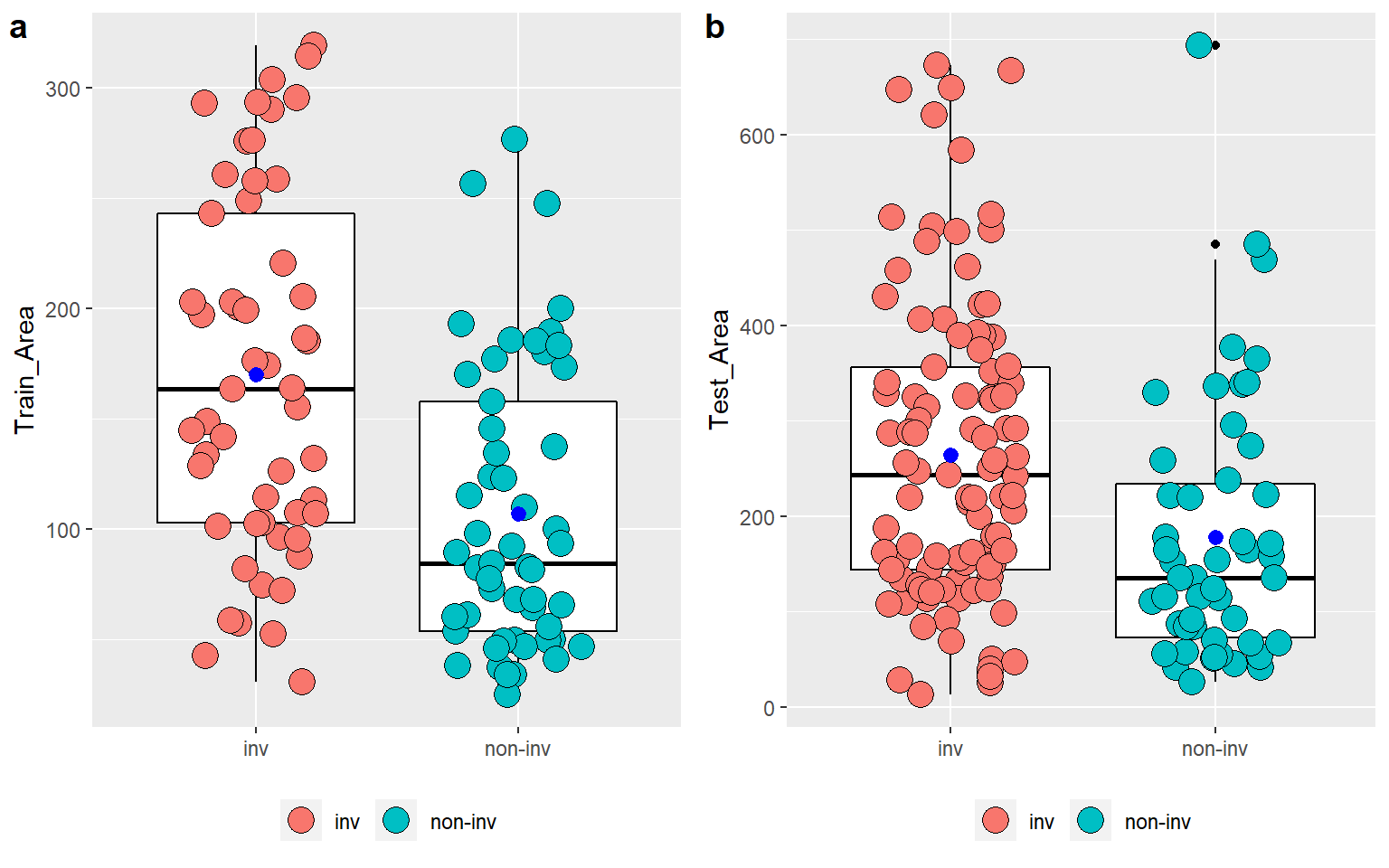


**S.Figure 3.** Boxplot for a) training and b) testing set for area of the nodule
